# Essential Oils from Ugandan Aromatic Medicinal Plants: Chemical Composition and Growth Inhibitory Effects on Oral Pathogens

**DOI:** 10.1155/2015/230832

**Published:** 2015-06-10

**Authors:** Francis Ocheng, Freddie Bwanga, Moses Joloba, Abier Softrata, Muhammad Azeem, Katrin Pütsep, Anna-Karin Borg-Karlson, Celestino Obua, Anders Gustafsson

**Affiliations:** ^1^Department of Dentistry, School of Health Sciences, College of Health Sciences, Makerere University, P.O. Box 7072, Kampala, Uganda; ^2^Department of Medical Microbiology, School of Biomedical Sciences, College of Health Sciences, Makerere University, P.O. Box 7072, Kampala, Uganda; ^3^Department of Dental Medicine, Unit of Periodontology, Karolinska Institutet, P.O. Box 4064, 141 04 Huddinge, Sweden; ^4^Ecological Chemistry Group, Department of Chemistry, School of Chemical Science and Engineering, KTH, Royal Institute of Technology, 100 44 Stockholm, Sweden; ^5^Department of Chemistry, COMSATS Institute of Information Technology, Abbottabad 22060, Pakistan; ^6^Department of Microbiology, Tumor and Cell Biology, Karolinska Institutet, P.O. Box 280, 177 71 Stockholm, Sweden; ^7^Department of Pharmacology and Therapeutics, School of Biomedical Sciences, College of Health Sciences, Makerere University, P.O. Box 7072, Kampala, Uganda

## Abstract

The study assessed the growth inhibitory effects of essential oils extracted from ten Ugandan medicinal plants (*Bidens pilosa, Helichrysum odoratissimum, Vernonia amygdalina, Hoslundia opposita, Ocimum gratissimum, Cymbopogon citratus, Cymbopogon nardus, Teclea nobilis, Zanthoxylum chalybeum,* and *Lantana trifolia*) used traditionally in the management of oral diseases against oral pathogens. Chemical compositions of the oils were explored by GC-MS. Inhibitory effects of the oils were assessed on periodontopathic *Porphyromonas gingivalis* and *Aggregatibacter actinomycetemcomitans* and cariogenic *Streptococcus mutans* and *Lactobacillus acidophilus* using broth dilution methods at concentrations of 1%, 0.1%, and 0.01%. The most sensitive organism was *A. actinomycetemcomitans*. Its growth was markedly inhibited by six of the oils at all the concentrations tested. Essential oil from *C. nardus* exhibited the highest activity with complete growth inhibition of *A. actinomycetemcomitans* and *P. gingivalis* at all the three concentrations tested, the major constituents in the oil being mainly oxygenated sesquiterpenes. Most of the oils exhibited limited effects on *L. acidophilus*. We conclude that essential oils from the studied plants show marked growth inhibitory effects on periodontopathic *A. actinomycetemcomitans* and *P. gingivalis*, moderate effects on cariogenic *S. mutans*, and the least effect on *L. acidophilus*. The present study constitutes a basis for further investigations and development of certain oils into alternative antiplaque agents.

## 1. Introduction

Dental caries (DC) and periodontal diseases (PD) are common oral conditions [1], caused by bacterial dental plaque in the oral cavity [2]. Cariogenic bacteria, such as* Streptococcus mutans*,* Streptococcus sobrinus*, and* Lactobacillus acidophilus*, as well as periodontopathic bacteria like* Porphyromonas gingivalis* and* Aggregatibacter actinomycetemcomitans* are associated with these oral conditions [3, 4].

Maintenance of effective oral hygiene through regular removal of accumulated plaque from tooth surfaces is one of the major approaches to reducing DC and PD [5]. Conventional tooth brushing is recommended and widely promoted for removal of accumulated plaque but data show that the vast majority of people are unable to accomplish this on a regular basis [6]. A number of mouthwash solutions have been introduced to produce inhibitory effects on plaque formation and act as adjuncts to tooth brushing [7, 8]. In general, most of these mouth rinses contain fluorides, alcohols, detergents, and synthetic antimicrobials, including iodine products, chlorhexidine, benzalkonium chloride, cetylpyridinium chloride, and triclosan [7, 8]. However, some synthetic mouth rinses, like chlorhexidine, are associated with staining of teeth [9] and others, like triclosan, have been shown to negatively affect environmental microbes and ecosystems [10]. This scenario has necessitated the search for new potential alternative antibacterial agents that can be incorporated in the mouth rinses.

Recently, there have been renewed interests in traditional medicinal natural products due to their availability, as well as better biodegradability compared to the synthetic agents [11]. Particularly, there has been increased interest looking at biological activities of essential oils of aromatics medicinal plants [11, 12]. Essential oils are to, a large extent, mixtures of terpenoids, specifically monoterpenes [C_10_] and sesquiterpenes [C_15_], although diterpenes [C_20_] may also be present, and a variety of low molecular weight aromatic and aliphatic alcohols, ethers, aldehydes, and ketones [13]. They have a number of potential uses, including food flavoring and preservation from spoilage [14] and pharmaceuticals, owing to their notable antioxidant [15] and antimicrobial [11, 16] attributes. Despite advances in research and application of essential oils in human health [12] there are few studies evaluating their use as alternatives to synthetic agents for the control of dental plaque [17, 18].

We previously investigated antibacterial activities of fresh pulp juice and solvent extracts obtained from 16 medicinal plants used in traditional management of various forms of oral diseases in Uganda [19]. From the original 16 plants species, ten were selected based on the findings of our previous study and their groupings in aromatic plants families [20]. The antibacterial activities of extracts from many of these plants have been investigated on other bacteria [21–23]. However, the inhibiting effects on periodontal pathogens have not been investigated and only some of the plants have been tested against bacteria associated with DC [24]. Therefore, the aim of the present study was to investigate the growth inhibitory effects of the essential oils extracted from the ten aromatic plants against a panel of Gram-negative bacteria associated with PD and Gram-positive bacteria associated with DC. Furthermore, we analyzed the chemical composition of the essential oils.

## 2. Materials and Methods

### 2.1. Plant Materials

The ten aromatic plants selected for extraction of essential oils were* Bidens pilosa*,* Helichrysum odoratissimum*,* Vernonia amygdalina*,* Hoslundia opposita*,* Ocimum gratissimum*,* Cymbopogon citratus*,* Cymbopogon nardus*,* Teclea nobilis*,* Zanthoxylum chalybeum*, and* Lantana trifolia*. The selection was done on the basis of their grouping in the aromatic plants families, namely, Asteraceae, Lamiaceae, Poaceae, Rutaceae, and Verbenaceae [20], and traditional use in the treatment of various forms of dental or oral diseases as reported in literature (Table 1). Ethnomedical information on the plants is summarized in Table 1.

Fresh leaves and twigs of the plants were collected from their natural habitats from different regions of Uganda during the second half of 2011. Identities of the plants were confirmed by botanists at the Herbarium, Department of Botany, Makerere University, Uganda, where voucher specimens were also archived.

### 2.2. Extraction of Essential Oils

To extract essential oils, 300 g of cut pieces of fresh plant materials was mixed with 600 mL of distilled water and the mixture subjected to hydrodistillation for 4-5 hours using glass distillation apparatus. The distillate (oil/water mixture) was collected and the essential oils were extracted from the distillate with HPLC grade hexane (VWR International, Sweden). Anhydrous magnesium sulfate (VWR International, Sweden) was added to the hexane extract to remove any trace of water. After filtration, hexane was evaporated with a rotary evaporator (Buchi Rotavapor R210, Switzerland). The essentials oils obtained were then weighed and the yields calculated as percentage of fresh starting plant materials.

### 2.3. Bacterial Strains and Culture Conditions

*Gram-Negative Bacterial Strains*. The Gram-negative bacterial strains were periodontopathic bacteria* Aggregatibacter actinomycetemcomitans* (HK 1519) and* Porphyromonas gingivalis* (ATCC 33277).

*Gram-Positive Bacterial Strains*. The Gram-positive bacterial strains were cariogenic bacteria* Streptococcus mutans* (CCUG 27624) and* Lactobacillus acidophilus* (NCTC 1723) and the nonoral pathogenic bacterium* Bacillus megaterium* (BM11).

*Growth Conditions*. All the bacteria were propagated as previously described [18]. Briefly,* A. actinomycetemcomitans* was propagated on Columbia base agar (Acumedia, Baltimore, MD, USA) supplemented with 0.1% tryptophan (Merck, VWR International, Sweden) and 5% citrated horse blood in 5% CO_2_ atmosphere (CampyPak, Becton Dickinson, Sweden).* P. gingivalis* was propagated for 6 days on Colombia base agar supplemented with hemin (0.05 mg/mL), vitamin K (0.01 mg/mL) (BBL, Becton Dickinson, Sweden), and 5% citrated horse blood in an anaerobic atmosphere (GasPak, Becton Dickinson, Sweden).* S. mutans* was grown in Brain-Heart Infusion (BHI) agar plates (Oxoid, Malmo, Sweden) for 2 days in 5% CO_2_ atmosphere.* L. acidophilus* was propagated for two days on Lactobacilli MRS agar plates (Difco, Becton Dickinson, Sweden) in 5% CO_2_.* B. megaterium* was propagated overnight in air on Luria Agar plates (Difco). All bacteria were incubated at 37°C.

### 2.4. Analysis of Chemical Composition of Essential Oils

The chemical composition of the essential oils was analyzed using a Varian 3400 Gas-Chromatography (GC) connected to a Finnigan SSQ 7000 Quadrupole Mass Spectrometer (MS). The GC was equipped with a split/splitless injector (splitless mode 30 seconds), a DB-wax capillary column (J&W Scientific, Folsom, CA, USA; 30 m length, 0.25 mm inner diameter, and 0.25 *μ*m film thickness). The injection temperature was isothermally set at 230°C. The carrier gas (helium, 99.99%, Stransmollen AB, Sweden) was delivered at a constant pressure of 10 psi. A representative temperature program was 40°C for 1 minute, followed by an increase in temperature at a rate of 3°C/minute up to 235°C, and thereafter the temperature was maintained at 235°C for 14 minutes, making up a total analysis time of 80 minutes. Transfer line temperature was kept at 235°C and the MS ion source temperature was 150°C. Mass spectra were obtained for 70 eV with a mass range of 30 to 600 *m*/*z* in positive mode. The software program X-calibur 2.0 was used for acquiring and analysis of the GC-MS data. For analysis, dried samples of essential oils were reconstituted in hexane to a concentration of 5 *μ*g/*μ*L and 1 *μ*L injected into the GC. Identification of compounds in the oils was made by comparison of their MS with compounds in the Finnigan NIST Library-2008 and final authentication of selected compounds made by analyzing available compounds at the same parameters as those used for the essential oils.

### 2.5. Assessment of Growth Inhibitory Effects of the Essential Oils

Assessment of growth inhibitory effects was done using broth dilution method as previously described [18], with minor modifications. Colonies of* A. actinomycetemcomitans* and* P. gingivalis* were resuspended in Peptone Yeast Glucose (PYG) medium.* S. mutans* colonies were resuspended in BHI broth. Colonies of* L. acidophilus* were resuspended in Lactobacilli MRS broth. Colonies of* B. megaterium* were resuspended in Luria broth. The optical densities of all bacterial suspensions were adjusted to 0.5 at 590 nm wavelength. All bacteria were further diluted in fresh growth medium 10^4^-fold prior to the test. The bacterial suspensions were incubated for 90 minutes in their respective growth media at 37°C in the presence of different concentrations of essentials oils. Dilutions of essential oils were prepared in dimethyl sulfoxide (DMSO) (Sigma-Aldrich, Sweden) and 5 *μ*L of each dilution or undiluted oil was added to 495 *μ*L of the respective growth medium to obtain final concentrations of oils in media 1%, 0.1%, and 0.01%. To the solvent control, 5 *μ*L of DMSO was added. For the positive control, chlorhexidine was used. It was diluted in media to get final concentrations of 1%, 0.02%, and 0.05% and tested against clinically relevant bacteria (*A. actinomycetemcomitans*,* P. gingivalis*,* S. mutans*, and* L. acidophilus*). After 90 minutes of incubation, the bacterial suspensions were evenly spread on agar plates with the respective growth media and propagated as described in Section 2.3. The numbers of live bacteria were determined by counting the colonies on each plate, which equal colony forming units (CFU). Growth inhibitory effects were expressed as the percentage of the colony forming units (CFU) in the presence of plant essential oils or chlorhexidine to the CFU in the control plate ((CFU in test/CFU in control) × 100). All tests were performed in duplicate and repeated twice.

### 2.6. Statistical Analysis

Differences in CFU in the control plate and CFU in each tested concentration of the oil or chlorhexidine (in the original dataset) were statistically analyzed using independent Student's *t*-test. Values of *p* < 0.05 were regarded as significant (*n* = 4).

## 3. Results and Discussion

### 3.1. Essential Oils Yields and Chemical Composition

The essential oils yields expressed in relation to fresh weight of plant materials (% w/w) are presented in Table 2. The yields varied from 0.05% to 0.39%. The highest (0.39% w/w) and the lowest (0.05% w/w) yield were obtained from* C. citratus* and* B. pilosa*, respectively. Differences in oil yields were seen in reports for the same aromatic plants species such as* T. nobilis *[30] and* C. citratus* and* C. nardus* [31] collected in other geographic areas in the world. This could be attributed to external factors such as climate, nature of the soil, age of the tree, and time of collection but also mode of extraction.

The chemical composition of the essential oils was analyzed by GC-MS. The major compounds which were 1% or larger in each oil are presented in Table 2. The most widely distributed compound was *β*-caryophyllene, a sesquiterpene hydrocarbon, which was found in all the oils except those extracted from* O. gratissimum, T. nobilis*, and* Z. chalybeum.*

### 3.2. Growth Inhibitory Effects of the Essential Oils

**Figure 1:**
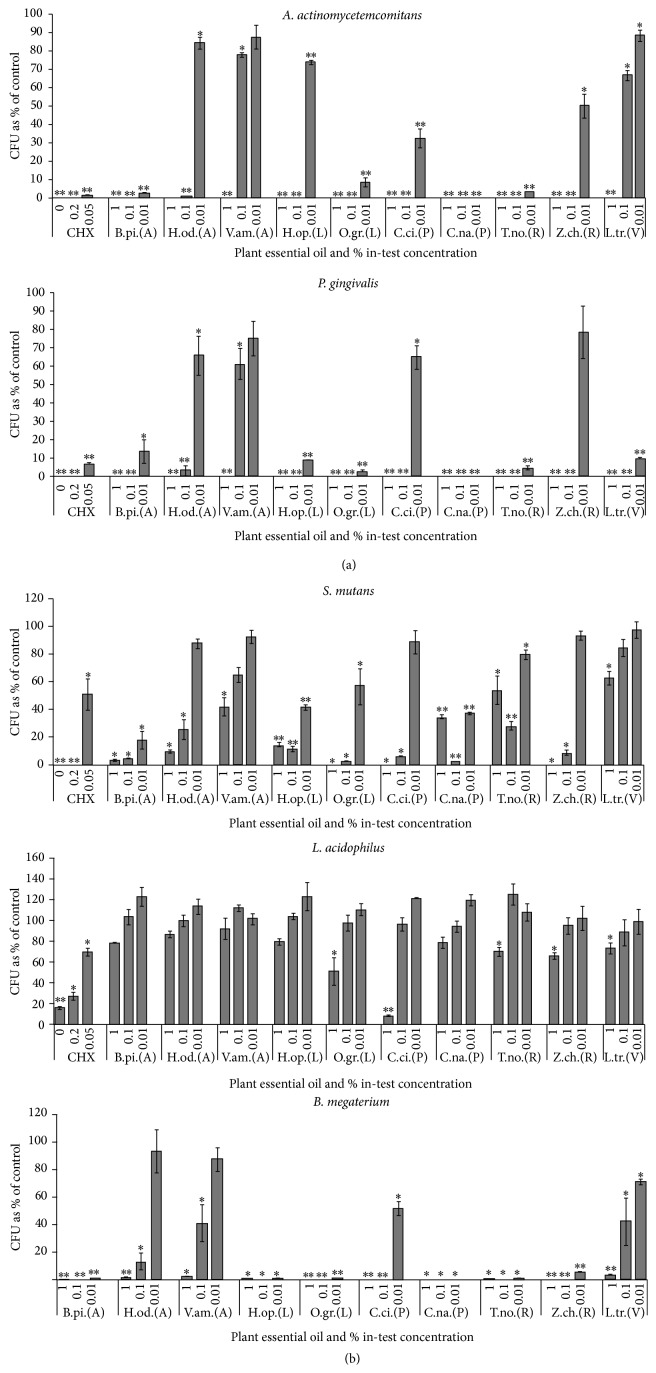
Inhibitory effects of plant essential oils on bacterial growth determined by colony forming units (CFU) assay. The individual bars show the number of surviving bacteria expressed as a percentage of control (*n* = 4, error bar = S.E.M). Differences in CFU in the control plate and CFU in each tested concentration of the oil or chlorhexidine (in the original dataset) statistically analyzed using independent Student's *t*-test: ^*∗*^ *p* < 0.05 to 0.0001, ^*∗∗*^ *p* < 0.0001 compared with the control. The plant essential oil in-test concentration is in percentage of final assay volume. (a) Gram-negative bacteria:* A. actinomycetemcomitans*,* P. gingivalis*. (b) Gram-positive bacteria:* S. mutans*, L.* acidophilus*,and* B. megaterium*. Positive control: CHX = chlorhexidine. Plant species names: B.pi =* Bidens pilosa*; H.od =* Helichrysum odoratissimum*; V.am =* Vernonia amygdalina*; H.op =* Hoslundia opposita*; O.gr =* Ocimum gratissimum*; C.ci =* Cymbopogon citratus*; C.na =* Cymbopogon nardus*; T.no =* Teclea nobilis* Delile; Z.ch =* Zanthoxylum chalybeum*; L.tr =* Lantana trifolia.* Plant family names: (A) = Asteraceae; (L) = Lamiaceae; (P) = Poaceae; (R) = Rutaceae; (V) = Verbenaceae.

The essential oils from the ten plants were assessed for their growth inhibitory effects on two Gram-negative periodontopathic bacteria and two Gram-positive cariogenic bacteria using broth dilution assay.* B. megaterium* (Gram-positive) was included in the tests to represent a nonoral pathogenic reference bacterium. This bacterium has been frequently used as a reference strain in studies of antibacterial activities of endogenous and exogenous substances [18, 32]. The growth inhibitory effects of the oils are presented in Figure 1. Generally most of the tested organisms were sensitive to many of the oils and the chlorhexidine positive control, apart from* L. acidophilus*, whose growth was not significantly inhibited by any of the oils. However, the growth of* L. acidophilus* continued to be inhibited by the chlorhexidine positive control at the three tested concentrations of 1%, 0.2%, and 0.05% with *p* values of <0.0001, 0.0001, and 0.0107, respectively (Figure 1(b)). The most sensitive organism was* A. actinomycetemcomitans*, as its growth was markedly inhibited by six of the plant oils and the chlorhexidine positive controls at all the three concentrations tested with *p* < 0.0001 (Figure 1(a)). This was followed by* P. gingivalis*, which was markedly inhibited by five of the oils and the chlorhexidine control at the three concentrations tested with *p* < 0.0001 (Figure 1(a)).

The traditional use and anecdotal evidence of plants as medicine provide the basis for suggesting that essential oils and other plant extracts may be useful for specific medical conditions. In light of this, our group assessed the essential oils from the Ugandan aromatic medicinal plants used in traditional management of oral/dental diseases for their growth inhibitory effects on oral pathogens. The results of this study showed that essential oils from the C.* nardus* plant exhibited the highest activity with complete growth inhibition of the oral pathogens* A. actinomycetemcomitans* and* P. gingivalis* and the nonoral pathogenic* B. megaterium* at all the three concentrations tested (Figures 1(a) and 1(b)). It also showed inhibition to the growth of* S. mutans* at all the three concentrations with *p* < 0.0001 (Figure 1(b)). Previous reports described essential oil from C.* nardus* to be bactericidal to the human pathogens,* E. coli* strain NCTC 10418, and* Staphylococcus aureus*, though at a concentration of 0.25% (v/v) [16]. GC-MS analysis revealed the major constituents in* C. nardus* oil to be dominated by oxygenated sesquiterpenes, specifically intermedeol (Table 2), and it is probable that these compounds could be responsible for the strong growth inhibitory effect observed.* C. citratus*, another member of the Poaceae family, was found to be rich in oxygenated monoterpenes, specifically geranial and neral. It gave rise to growth inhibition of* A. actinomycetemcomitans* at 1%, 0.1%, and 0.01% concentrations,* P. gingivalis* and* B. megaterium* at 1% and 0.1% concentrations, and* L acidophilus* at 1% concentration, with *p* < 0.0001.* C. citratus* also showed inhibition to the growth of* S. mutans* at concentrations of 1% and 0.1% with *p* values of 0.0005 and 0.0007, respectively. The finding is in line with previous studies where essential oil from* C. citratus* was found to inhibit the growth of cariogenic* S. mutans* at concentration of 250–500 *μ*g/mL [33] and several* Helicobacter pylori* strains at a concentration of 0.01% (v/v) [34]. The activity of* C. citratus* is attributed to generial and neral (collectively known as citral), which have been reported to possess the most significant antimicrobial activity amongst the constituents [35]. The higher potency could be related to the high lipophilicity of citral (aldehyde), which enhances its interaction with bacterial cell membranes thereby inducing higher damage [36].

Essential oils from plants belonging to the Lamiaceae family also demonstrated very high activity. The oil from* H. opposita* containing sesquiterpene hydrocarbons, such as *β*-caryophyllene, germacrene D, humulene, and *α*-cadinol, was especially effective on the clinically relevant* A. actinomycetemcomitans*,* P. gingivalis*, and* S. mutans* as their growth was markedly inhibited by this oil at all the three concentrations (*p* < 0.0001) (Figures 1(a) and 1(b)). This finding agrees with previous studies where the essential oil extracted from* H. opposita* was found to contain mainly sesquiterpenes and sesquiterpene alcohols with significant activity against* Aspergillus niger*,* Acinetobacter calcoaceticus*,* Brochothrix thermosphacta*, and* Flavobacterium suaveolens* [21]. The major compound in the oil of* O. gratissimum* was an aromatic compound, eugenol (Table 2). This compound is known for its antimicrobial activity [37] and is widely used in dentistry.

Essential oil from* T. nobilis* was the most active among the Rutaceae family tested, as it markedly inhibited growth of* A. actinomycetemcomitans* and* P. gingivalis* at the three concentrations (*p* < 0.0001) (Figure 1(a)). It was also inhibitory to* S. mutans* at concentrations of 1%, 0.1%, and 0.01% with *p* values of 0.0001, <0.0001, and 0.0003, respectively, and* B. megaterium* at all the three concentrations with *p* values of 0.0005 (Figure 1(b)). The oil was found to be rich in sesquiterpenes, specifically germacrene D (Table 2). The finding is contrary to previous studies, in which no antimicrobial activity was exhibited by the oil [30]. The plants in that study were collected from Southern province of Saudi Arabia with major compounds containing mainly mixtures of monoterpenes and sesquiterpenes [30]. The chemical composition of essential oils may change according to the habitat (chemotypes) and the time point at which the plants are harvested, plant growth phase, which in turn influence their antimicrobial capacity [14]. The essential oil from* Z. chalybeum*, the other member of Rutaceae family, markedly inhibited growth of* A. actinomycetemcomitans* and* P. gingivalis,* at 1% and 0.1% concentrations (*p* < 0.0001) (Figure 1(a)). It was highly effective on the nonoral pathogenic bacterium* B. megaterium* at all the three concentrations (*p* < 0.0001) (Figure 1(b)). GC-MS analysis revealed the major constituent in the oil of* Z. chalybeum* essential oil to be an oxygenated monoterpene, terpinene-4-ol, which is a known antimicrobial compound [38]. To the best of our knowledge, this is the first report on the antibacterial effects of essential oil from* Z. chalybeum*.

Essential oil from* B. pilosa* demonstrated the best activity among the three Asteraceae species tested. It markedly inhibited growth of* A. actinomycetemcomitans* and* B. megaterium* at all the three concentrations and* P. gingivalis* at 1% and 0.1% concentrations with *p* < 0.0001. The oil also inhibited growth of* S. mutans* at 1%, 0.1%, and 0.01% concentrations with *p* values of 0.0005, 0.0006, and, 0.0013, respectively. Other studies have confirmed antimicrobial activities in* B. pilosa* essential oil and *β*-caryophyllene as a major compound [39].

Most of the essential oils in this study exhibited limited effects on the growth of Gram-positive oral pathogens* L. acidophilus* (Figure 1(b)). In previous works, essential oil or volatiles from the root of* Salvadora persica* were also found to have limited or no effect on* L. acidophilus* [18, 40]. This inherent resistance of lactobacillispp. to some antimicrobial agents has been suggested to be due to the absence of hydrogenase activity [41].

An important characteristic of essential oils and/or their components is their hydrophobicity [14]. This characteristic enables the essential oil to be partitioned in the lipids of the bacterial cell membrane, disturbing the structures and rendering them more permeable. Leakage of ions and other cell contents occurs leading to bacterial cell death.

Previously, using transmission electron microscopy (TEM), we demonstrated evidence of interference with the bacterial envelope leading to bacterial membrane protrusions and cell death by the essential oil from* Salvadora persica* root, with the main component benzyl isothiocyanate [18]. Devi and coworkers [37] observed the effect of eugenol, a major component of* O. gratissimum*, on* Salmonella *Typhi cell surface by scanning electron microscopy (SEM), and Tyagi and Malik [42], using SEM, observed that* Escherichia coli* cells, treated with* Cymbopogon citratus*, appeared to be aggregated and partially deformed.

Collectively, these morphological and ultrastructural observations provide evidence that essential oils and/or components have the capability to alter cell permeability by entering between the fatty acyl chains, thus interfering with membrane lipid bilayers of bacterial cells and disrupting the lipid packing, which eventually results in complete loss of its integrity [36]. However, considering the large number of different chemical groups and large variety of molecular structures in the presently investigated essential oils, it is most likely that the activity is not attributable to one specific compound or mechanism but instead there are several targets in the bacterial cell [43]. Future studies using isolated compounds and combinations of these compounds will give insights into the antibacterial mechanisms of the various plant essential oils.

Periodontopathic and cariogenic bacteria are clinically present in the dental plaque as a biofilm [2]. Increased understanding of biofilm characteristics has demonstrated that there are differences between bacterial behaviors in laboratory culture and in their natural ecosystems [2]. More studies of the effect of these essential oils on the bacteria in their natural ecosystem should give more insights for further developments of the oils into useable alternative plant-based products.

## 4. Conclusion

In conclusion, most of the oils in this study showed marked growth inhibitory effects on the clinically relevant periodontopathic bacteria* A. actinomycetemcomitans* and* P. gingivalis*, moderate inhibitory effect on cariogenic* S. mutans*, and least effect on* L. acidophilus*. The most promising essential oils were from* C. nardus*,* T. nobilis*,* H. opposita*,* O. gratissimum*, and* B. pilosa* as they markedly inhibited growth of at least two bacteria at all the three concentrations tested with *p* < 0.0001. Thus, the present study may constitute a basis for further investigation and development of these oils into alternative antiplaque agents.

## Figures and Tables

**Table 1:** Aromatic medicinal plants used in traditional treatment of oral diseases in Uganda.

Family	Species name (voucher number)^a^	Plant part: ethnomedical use(s) [references]	Oral disease(s) treated
Asteraceae	*Bidens pilosa* (FO-002)	L: chew [25]	Toothache

Asteraceae	*Helichrysum odoratissimum* (FO-004)	L: dry, burn, and rub ash on false teeth [25]	Teething syndrome

Asteraceae	*Vernonia amygdalina* (FO-005)	S: brush teeth, twigs chewed [25, 26]	Dental caries

Lamiaceae	*Hoslundia opposita* (FO-008)	L: chewed [27, 28].	Mouth wounds

Lamiaceae	*Ocimum gratissimum* (FO-009)	L: chewed [25]	Toothache

Poaceae	*Cymbopogon citratus* (FO-010)	L: taken as tea; chewed fresh [26]	Bad breath, toothache

Poaceae	*Cymbopogon nardus* (FO-011)	R: chewed; L: young part chewed and used for cleaning [25]	Bad breath, dental caries

Rutaceae	*Teclea nobilis* Delile (FO-013)	S: used to brush teeth [27]	Dental caries

Rutaceae	*Zanthoxylum chalybeum* (FO-014)	R: used to brush; S: bark chewed [28]	Dental caries Toothache

Verbenaceae	*Lantana trifolia* (FO-016)	S: used to brush teeth; L: infusion swallowed [28, 29]	Oral hygiene Tonsillitis

^a^Voucher specimen number at the Herbarium, Department of Botany, Makerere University.

L: leave; S: stem; R: root.

**Table 2:** Chemical composition of the essential oil obtained from the aromatic medicinal plants.

Name of constituent	Percentage of constituents in essential oil^a^									
	[A]^b^	[A]	[A]	[L]	[L]	[P]	[P]	[R]	[R]	[V]
	B.pi^c^	H.od	V.am	H.op	O.gr	C.ci	C.na	T.no	Z.ch	L.tr
	(0.05)^d^	(0.31)	(ND)	(0.12)	(0.21)	(0.39)	(0.36)	(0.16)	(0.21)	(0.14)
*Monoterpenes *										
3-Carene									8.3	
4-Carene									2.8	
Limonene									2.5	
Myrcene						10.2	10.5			
*cis-β*-Ocimene					3.7				2.1	
*trans*-*β*-Ocimene			3.8		7.6			8.5		
*α*-Phellandrene									5.1	
*β*-Phellandrene									1.5	
*α*-Pinene		4.2							1.1	
*β*-Pinene									2.6	3.0
Terpinolene									1.4	
*Oxygenated monoterpenes *										
Artemiseole						1.6				
1,8-Cineol									1.1	
Citronellal									1.1	
Geranial						35.7			13.3	
Geranic acid						7.2				
Geraniol						3.8			2.4	
Geranyl acetate									1.5	
Linalool	1.4				1.0	1.3			6.4	
Neral						28.6			9.9	
Nerolic acid						2.5				
Terpinene-4-ol									22.3	
*α*-Terpineol									2.2	
*cis-β-*Terpineol			4.0						1.2	2.4
*Sesquiterpenes *										
*α*-Bulnesene		2.8								
Cadinene	3.8				1.7					
*δ*-Cadinene		7.0		11.4				7.3		
Calarene							4.6			
*β*-Caryophyllene	12.6	12.6	5.9	10	3.5		1.0			8.4
Cedrene	1.5		3.0							3.3
*α*-Copaene		7.3		4.6	2.0					
*β*-Cubebene	11.7				10.9					
*γ*-Elemene							1.6	2.4		1.5
*β*-Elemene							1.9			
*E,E-α*-Farnesene			3.6							4.9
*β*-Farnesene					5.5					1.2
Germacrene D			27.5	28.7			1.3	54.4		23.7
*α*-Gurjunene								4.9		
Humulene		14.1		24.4						2.9
*α*-Muurolene							4.3			
*β*-Patchoulene							4			
Selina-3,7(11)-diene		3.3								
Thujopsene	4.6									
*Oxygenated sesquiterpenes *										
*α*-Cadinol	1.3			1.8			2.1	9.1		1.2
Tau-Cadinol								2.0		
Elemol							1.8			
Eudesmol							1.8			
Germacrene D-4-ol							8.6			
*(−)*-Globulol	1.0									
Guaiol							1.2			
Intermedeol							43.7			
Ledol					1.2					
Levomenol		7.3								
Nerolidol	1.1		2.4					1.9		
3-Methyl-4-(1,3,3-trimethyl-7-oxa-bicyclo[4.1.0]hept-2-yl)-but-3-en-2-one							3.4			
Muurolol								3.4		
Diterpene										
Phytol		1.6	2.3	4.3				1.2		5.9
*Aromatic compounds *										
Elixene	5.1									
Eugenol			18.3		56.4					3.7
Methyl isoeugenol								1.7		
Aromatic compound	24.4									
*Aliphatic compounds *										
Decanal									1.1	
19,19-Dimethyl-eicosa-8,11-dienoic acid		3.8								
3,4-Dimethyl-1-hexene			2.4							1.1
Ethyl linolenate		3.9		3.7						18.4
Methyl linolenate										3.1
1-Hexanol	2.2									
2-Hexen-1-ol	1.2									
2-Hexenal	1.6									
6-Methyl-3-heptanol										2.7
6-Methyl-5-hepten-2-one						1.5				
Methyl octadec-9-en-12-ynoate		2.2								
Myristic acid		1.4								
3-Octanol			3.9							
1-Octen-3-ol			13.9		1.1					
Palmitic acid		27.1	5.9	10.2				2.1		11.2
2-Undecanone						1.1				
Others^e^	26.5	1.4	3.1	1.0	5.4	6.5	8.2	1.1	10.1	1.4

^a^Expressed as percentage of the peak area relative to the total peak area and only constituents which were 1% or larger are shown.

^b^Plant family names: [A] = Asteraceae; [L] = Lamiaceae; [P] = Poaceae; [R] = Rutaceae; [V] = Verbenaceae.

^c^Plant species names: B.pi = *Bidens pilosa*; H.od = *Helichrysum odoratissimum*; V.am = *Vernonia amygdalina*; H.op = *Hoslundia opposita*; O.gr = *Ocimum gratissimum*; C.ci = *Cymbopogon citratus*; C.na = *Cymbopogon nardus*; T.no = *Teclea nobilis* Delile; Z.ch = *Zanthoxylum chalybeum*; L.tr = *Lantana trifolia*.

^d^Essential oil yield (% w/w); ND = yield not determined.

^e^Other compounds which were less than 1% in the oil.
